# Natural Phytochemicals in the Treatment and Prevention of Dementia: An Overview

**DOI:** 10.3390/molecules21040518

**Published:** 2016-04-21

**Authors:** Rosaliana Libro, Sabrina Giacoppo, Thangavelu Soundara Rajan, Placido Bramanti, Emanuela Mazzon

**Affiliations:** IRCCS Centro Neurolesi “Bonino-Pulejo”, Via Provinciale Palermo, Contrada Casazza, 98124 Messina, Italy; rosalianalibro@hotmail.it (R.L.); giacoppo.sabrina@hotmail.it (S.G.); tsrajanpillai@gmail.com (T.S.R.); bramanti.dino@gmail.com (P.B.)

**Keywords:** dementia, phytochemicals, polyphenols, isothiocyanates, alkaloids, cannabinoids

## Abstract

The word dementia describes a class of heterogeneous diseases which etiopathogenetic mechanisms are not well understood. There are different types of dementia, among which, Alzheimer’s disease (AD), vascular dementia (VaD), dementia with Lewy bodies (DLB) and frontotemporal dementia (FTD) are the more common. Currently approved pharmacological treatments for most forms of dementia seem to act only on symptoms without having profound disease-modifying effects. Thus, alternative strategies capable of preventing the progressive loss of specific neuronal populations are urgently required. In particular, the attention of researchers has been focused on phytochemical compounds that have shown antioxidative, anti-amyloidogenic, anti-inflammatory and anti-apoptotic properties and that could represent important resources in the discovery of drug candidates against dementia. In this review, we summarize the neuroprotective effects of the main phytochemicals belonging to the polyphenol, isothiocyanate, alkaloid and cannabinoid families in the prevention and treatment of the most common kinds of dementia. We believe that natural phytochemicals may represent a promising sources of alternative medicine, at least in association with therapies approved to date for dementia.

## 1. Introduction

### The Etiopathogenesis of Dementia

Dementia is an age-related irreversible condition resulting in a progressive cognitive decline that reduces a person’s ability to perform daily activities. Despite the progress made in the field of dementia in the last decades, the precise pathogenetic mechanisms of dementia are still not well understood. Dementia affects nearly 47.5 million patients worldwide and its incidence is predicted to increase significantly in the next decades since the average age of the population is increasing [1]. There are many different forms of dementia classified by the National Institute of Health: Alzheimer’s disease (AD), vascular dementia (VaD), dementia with Lewy bodies (DLB), frontotemporal dementia (FTD), and mixed dementias [2].

AD is the most common form of dementia worldwide, accounting for approximately 60% of all dementia cases, followed by VaD (20%), DLB (10%) and FTD (2%) [3]. AD is characterized by a gradual degeneration of the cholinergic neurons, in particular in the hippocampus and cortex areas that imply a loss of cognitive function causing symptoms such as memory loss, impaired judgement, depression and mental deterioration. The main pathological hallmarks of AD, including senile plaques, resulted from the extracellular accumulation of the amyloid beta (Aβ) protein, and the neurofibrillary tangles (NFTs), formed by hyperphosphorylated and aggregated Tau protein [4]. Aβ accumulation generates a cascade of events including oxidative stress and inflammation [5]. Furthermore, microglia activated by Aβ release pro-inflammatory cytokines, reactive oxygen species (ROS) and reactive nitrogen species (RNS), which cause mitochondrial dysfunction, leading to glutamate release and excitotoxic neuronal death. Additionally, NFTs form insoluble filaments that limit the transportation of neurotransmitters like acetylcholine (ACh) and interfere with communication between neurons contributing with Aβ oligomers to affect synaptic transmission, leading to cognitive impairment. Conventional therapies for AD are mainly symptomatic and consist of acetylcholinesterase inhibitors (AChEIs), among which donepezil (Aricept^®^), rivastigmine (RIV, Exelon^®^) and galantamine (GAL, Reminyl^®^) are widely used in AD patients. AChEIs enhance cholinergic transmission and show modest but statistically significant improvements on cognition and global functioning in mild to moderate AD [6]. To date another treatment recognized for moderate to severe AD is memantine (Namenda**^®^**), an antagonist of the *N*-methyl-d-aspartate (NMDA) receptor that has proven beneficial effects on the cognition, behavior and activities of daily living of AD patients [7].

VaD refers to a whole spectrum of cognitive dysfunctions, ranging from mild cognitive impairment to more severe cases that are characterized by a cerebrovascular etiology (cerebral ischemia, stroke). Reduced blood flow in the brain generates hypoxia and oxidative stress that trigger inflammatory responses and damage endothelial vessels, glial and neuronal cells [8]. In addition, cholinergic deficits have been reported in VaD patients. Although cholinergic therapies have shown promising effects on cognitive improvement [9], until now these treatments have not been validated for VaD. Current treatment approaches for VaD are aimed at preventing future vascular insults by controlling the major risk factors such as hypertension, hypercholesterolemia and diabetes mellitus [10].

DLB is a neurodegenerative dementia that generally occurs during the course of Parkinson’s disease, characterized by the abnormal aggregation of the α-synuclein (α-Syn) protein in neuronal cells, known as Lewy bodies [11]. The pathogenetic mechanisms involved in DLB are multifactorial, although genetic mutations in the α-Syn family genes have been implicated in the formation of Lewy bodies [12]. Clinically, DLB is characterized by cognitive decline, fluctuations in alertness and cognition, recurrent visual hallucinations, sleep disturbances, slowed movements, stiff limbs, and tremors (Parkinsonism). Neurodegeneration associated with DLB involves multiple brain areas including both dopaminergic and cholinergic neurons and for these reasons, it is often misdiagnosed as AD or other forms of dementia. Moreover, oxidative stress is significantly involved in the pathology of DLB [13]. In particular, α-Syn accumulation causes mitochondrial degeneration, which leads to the induction of oxidative stress followed by neurodegeneration. Current DLB therapies are directed at alleviating the symptoms and consist of drugs that restore dopamine signaling, such as levodopa, dopamine agonists and dopamine reuptake inhibitors [14].

FTD is a dementia characterized by early onset, and thus considered a dementia of the presenile age (<65 years of age). FTD is genetically and pathologically heterogeneous, characterized by progressive atrophy in the frontal or temporal lobes resulting in a gradual and progressive decline in behavior or language. In addition, neurovascular dysfunction contributes to FTD [1]. However, therapies for FTD are still missing and antipsychotics or antidepressants are typically administered to manage the symptoms [15].

The exact etiopathogenetic mechanisms leading to dementia have not yet been completely identified and the ongoing therapeutic strategies are generally based on the different aspects of dementia: to reduce protein aggregation, including β-amyloidosis and abnormal Tau phosphorylation in AD, and α-Syn deposition in DLB; to prevent further cerebrovascular and ischemic events in VaD and FTD; to restore specific neurotransmitter impairment, including cholinergic abnormalities in AD, and dysfunction of glutamatergic and dopaminergic system in DLB.

As already cited, conventional drugs used for most forms of dementia seem to act solely on symptoms, without having any profound disease-modifying effects. Although such treatments are effective in the early stages of the disease, long-term therapy has been associated with serious adverse effects [16,17]. Moreover, given the involvement of Aβ-induced oxidative stress in the etiology and pathology of dementia, one of the promising approaches of preventive interventions for dementia may be represented by antioxidant therapy which inhibits the detrimental effects of excess ROS through induction of endogenous antioxidant enzymes. Over the last decade, in an attempt to discover new alternative therapies for the most common form of dementia, basic science has focused on the discovery of natural compounds as potential candidates that can protect neurons against various insults and exert beneficial effects on neuronal cells. It is very likely that a dietary intake of foods or plant-based extracts with antioxidant as well as anti-inflammatory properties might have beneficial effects on human health and improve brain functions.

This review summarizes and discusses major *in vitro/in vivo* studies and clinical data demonstrating the neuroprotective effects of the most common natural phytochemicals belonging to the polyphenol, isothiocyanate, alkaloid and cannabinoid families in the prevention and/or in the treatment of the most common forms of dementia.

## 2. Polyphenols

Polyphenols are a class of natural compounds found mainly in fruits, vegetables, cereals and beverages, and considered the most abundant dietary antioxidants with an average consumption of around 1 g/day per person [18]. Polyphenol compounds can be classified into two main groups: non-flavonoids and flavonoids. More than 8000 phenolic structures are currently known and among them, more than 4000 flavonoids have been identified [19]. Non-flavonoid compounds include phenolic acids, stilbenes, lignans and other polyphenols (Table 1) [20]. Flavonoids are classified into six subgroups: flavones, flavonols, flavanols, flavanones, isoflavones, and anthocyanins [21].

The first evidence of the beneficial role of polyphenols in human health came from investigations in the 1960s and 1970s [22,23]. Further epidemiological studies have indicated that polyphenol consumption can be associated with a decreased risk to develop cancer [24], cardiovascular diseases [25] and neurodegenerative disorders [26]. Over the last decade, polyphenols have been suggested in the prevention and treatment of cognitive diseases, due to their antioxidative and anti-amyloidogenic features [27,28].

We performed a literature search using PubMed to identify articles about polyphenols and dementia, and found three most investigated polyphenols. By using the keywords “curcumin and dementia” 225 publications were found; by “resveratrol and dementia” 109 publications were found; and by searching for “epigallocatechin 3-gallate and dementia” 61 publications were found.

### 2.1. Curcumin: A Non-Flavonoid

**Figure 1:**
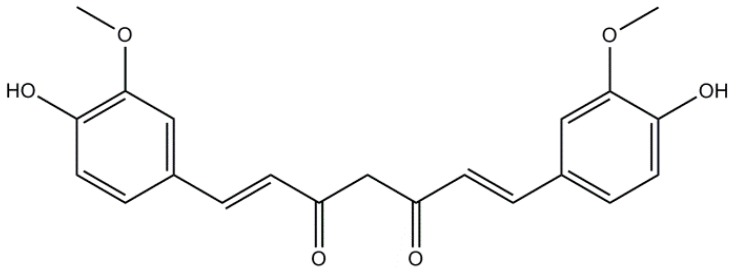
Molecular structure of curcumin.

Curcumin (CUR) or diferuloylmethane is extracted from *Curcuma longa*, a member of the ginger family, used for centuries in traditional Indian and Chinese medicine as a herbal remedy to cure inflammation of the skin and muscles [29]. The observation that Indian people aged 70–79 years consuming a diet rich in CUR had an incidence about 4.4-fold lower to develop AD than American people of the same age [30], led us to suppose that CUR could exert a neuroprotective role [31]. Indeed, numerous studies suggest CUR as a promising candidate for dementia therapy due to its neuroprotective activities including antioxidative, anti-inflammatory and anti-amyloidogenic effects [32,33]. The antioxidant properties of CUR are ascribed mainly to the presence of a phenolic group attached to two methoxy groups (Figure 1), which confers CUR the ability to transfer hydrogen atoms or sequentially transfer an electron and a proton [34]. CUR can scavenge hydroxyl and superoxide radicals *in vitro* and its antioxidant activity is considered to be around fourfold higher than α-tocopherol, a form of vitamin E [35]*.* CUR can act also as metal-chelator *in vivo* by binding with the redox-active metals iron and copper, and prevents neuroinflammation via metal induction inhibition of the Nuclear Factor Kappa B (NFκB) pathway in the brain of AD animal models [36].

Jin *et al.* [37] investigated the effect of CUR pre-treatment in lipopolysaccharide (LPS)-stimulated BV2 microglia cells. They found that CUR prevented the increased expression of inducible nitric oxide synthase (iNOS) and cyclooxygenase 2 (COX-2) which inhibited the consequent production of nitric oxide (NO) and prostaglandin E2 (PGE2), respectively. Moreover, CUR reduced the transcription levels of the pro-inflammatory cytokines interleukin-1beta (IL-1β), interleukin-6 (IL-6), and Tumor Necrosis Factor-alpha (TNF-α) by NFκB signaling inhibition. Similar results by Shi *et al.* [38] demonstrated that CUR protected mouse primary microglia cells from Aβ-toxicity in a dose-dependent manner by attenuating the release of IL-β, IL-6 and TNF-α via p38 mitogen-activated protein kinase (MAPK) and extracellular-signal-regulated kinases (ERK) inhibition. Parallel to these *in vitro* studies, CUR-mediated anti-inflammatory effects have been reported in *in vivo* models. The effect of CUR supplementation in diet at low (160 ppm) or at high doses (5000 ppm) for 6 months was investigated in Tg2576, an AD transgenic mouse model by Lim *et al.* [39]. The authors found that both doses of CUR decreased the expression of the pro-inflammatory cytokines, such as IL-1β, that was elevated in Tg2576 brains, as well as reduced the levels of oxidized proteins. Furthermore, they observed that animal treated with CUR at low doses showed a reduction of both insoluble amyloid and plaque burden as well as reduced levels of the glial fibrillary acidic protein (GFAP), a well-known marker of activated astrocytes. Rinwa *et al.* [40] investigated the effect of daily administration of CUR (20 mg/kg for 14 days) in another AD mouse model obtained by intracerebroventricular (icv) administration of streptozocin (STZ) (icv-STZ mouse). They found that CUR supplementation in this model reduced memory deficits by decreasing oxidative stress and AChE activity. In addition, they investigated the role of peroxisome proliferator-activated receptor gamma (PPAR-γ), an important negative regulator of inflammation [41], in CUR-stimulated anti-inflammatory effects. They found that icv-STZ AD mice pretreated with PPARγ antagonist failed to show the protective effect of CUR, suggesting a crucial role of PPARγ receptor in CUR-triggered anti-inflammatory effects [40].

Furthermore, several *in vitro* and *in vivo* studies highlighted the anti-amyloidogenic properties of CUR. Park and coauthors [42] reported that CUR pre-treatment (10 μg/mL for 1 h) reduced oxidative stress, intracellular calcium influx, and Tau hyperphosphorylation induced by Aβ exposure in rat pheocromocytoma PC12 cells. In human neuroblastoma cells SH-SY5Y expressing the Swedish mutant of the Amyloid Precursor Protein (APP_swe_), CUR treatment significantly reduced Aβ production in a dose- and time-dependent manner and this Aβ reduction was mediated by serine 9 residue phosphorylation of Glycogen Synthase Kinase 3 (GSK3β), a key enzyme involved in the phosphorylation of the Amyloid Precursor Protein (APP) and Tau proteins [43]. In murine neuroblastoma cells Neuro2a overexpressing the mutant APP_swe_ (N2a/APP_swe_), CUR treatment decreased the expression of presenilin-1 (PS1; γ-secretase) and beta-site amyloid precursor protein cleaving enzyme 1 (BACE-1; β-secretase), proteases involved in the synthesis of Aβ plaques [44].

Indeed, similar anti-amyloidogenic feature of CUR has been also demonstrated in *in vivo* models. 1,7-Bis(4′-hydroxy-3′-trifluoromethoxyphenyl)4-methoxycarbonylethyl-1,6-heptadiene-3,5-dione) (FMeC1), a novel curcumin derivative, significantly decreased the insoluble Aβ deposits, glial activation, and ameliorated the cognitive deficits in APP/PS1 double transgenic AD mice [45]. Another interesting study performed by Wang *et al.* [46] demonstrated that CUR may exert anti-amyloidogenic effects by inhibiting Phosphatidylinositol 3-Kinase (PI3K), phosphorylated protein kinase B (Akt) and mammalian target of rapamycin (mTOR) pathway (PI3K/Akt/mTOR pathway)-mediated formation of Aβ deposits in APP/PS1 AD mice [46]. Moreover, enzymes required for Aβ degradation, such as insulin-degrading enzymes and neprilysin, were found to be increased in these mice administered with CUR, which eventually improved the spatial learning and memory abilities [47]. Data reported by Garcia-Alloza *et al.* [48] provided further evidences for the anti-amyloidogenic effect of CUR. They showed that CUR crossed the blood brain barrier (BBB) and label Aβ, which eventually causes Aβ degradation in APP/PS1 AD mice. Furthermore, *in vivo* administration of CUR was shown to reduce high-cholesterol, a well-known risk factor for VaD and AD [49]. Tian *et al.* [50] showed that CUR administration lowered the cholesterol levels and ameliorated the vascular cognitive impairment in rat with chronic cerebral hypoperfusion (CCH), a VaD model [51,52]. They found that CUR decreased cholesterol levels by inducing the expression of the ATP-binding cassette transporter and apolipoprotein A1, which mediate cholesterol transmembrane transportation. The summary of molecular mechanisms underlying CUR-induced antioxidative, anti-inflammatory and anti-amyloidogenic effects discussed above is listed in Table 2.

Significant preclinical data obtained from *in vitro* and *in vivo* studies made clinicians to explore the therapeutic efficacy of CUR in dementia patients [53,54,55,56,57]. However, these clinical trials have failed to produce any convincing protection in AD patients. Possible reasons behind the unsuccessful results of these clinical trials are: (1) the molecular pathology underlying animal models with dementia is not same as that of humans; (2) the metabolism of CUR in rodents and in humans may differ.

Besides, the role of CUR in VaD, DLB and FTD patients is yet to be investigated. In summary, we propose the urgent need of compelling animal models of dementia, which reflect the similar pathology in dementia patients in order to successfully evaluate the therapeutic efficacy of CUR.

### 2.2. Resveratrol: A Non-Flavonoid

**Figure 2:**
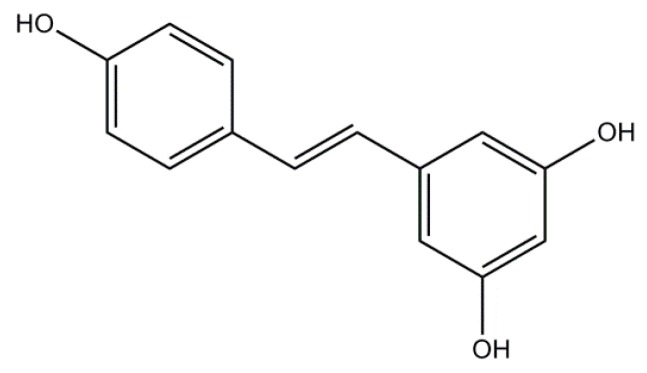
Molecular structure of resveratrol.

Resveratrol (RESV) belongs to a non-flavonoids class of polyphenolic compounds, called stilbenes, found in more than 70 different plants [58], including gnetum, butterfly orchid tree, white hellebore, Scots pine, corn lily, eucalyptus, spruce, and also in a lot of fruits and beverages, including grapes, cranberry, and wine. RESV is a phytoalexin synthesized from plants after exposure to stress, such as injury, fungal infections and UV radiation [58]. RESV can cross the BBB and produce neuroprotective effects against cerebral injury [59]. Structural studies demonstrated that the antioxidant properties of RESV depend on the presence of three hydroxyl groups in positions 3, 4 and 5 attached to the aromatic rings that offer RESV the ability to remove free radical species [60] (Figure 2). The antioxidant properties of RESV have been associated also with its ability to stimulate the expression of endogenous antioxidant enzymes. In healthy rats, RESV administration increased the activity of some detoxifying enzymes, such as superoxide dismutase (SOD) and catalase (CAT), while decreasing the activity of the pro-oxidant enzyme malondialdehyde (MDA) in mouse brain [61].

Many *in vitro* and *in vivo* studies have demonstrated the therapeutic efficacy of RESV in dementia models associated with AD. Kim *et al.* [62] found that pre-incubation with RESV (20 μM) in rat C6 glioma cells protected them against Aβ toxicity, by inhibiting iNOS and COX-2 expression and consequently reducing the production of PGE2 and NO. In PC12 cells exposed to Aβ toxicity, RESV pre-treatment (25 μM) protected cells against Aβ-induced oxidative cell death, by decreasing ROS accumulation, by attenuating the increased expression of pro-apoptotic proteins such as the Bcl-2-associated X protein (Bax), and by blocking the activation of the c-Jun N-terminal kinases (JNK) and NFκB [63]. Han *et al.* [64] showed that RESV treatment in rat hippocampal cells attenuated Aβ-induced cell-death in a concentration-dependent manner. Furthermore, they demonstrated that cells pre-treated with the protein kinase C (PKC) inhibitor significantly reduced the neuroprotective effect of RESV, suggesting the role of PKC in RESV-mediated neuroprotection [65]. RESV treatment in HEK293 and Neuro2a cells transfected with APP_swe_ variant attenuated Aβ accumulation by activating 5′ adenosine monophosphate-activated protein kinase (AMPK), a crucial regulator of cellular energy metabolism [66]. AMPK activation inhibits mTOR signaling and promotes autophagy and lysosomal degradation of Aβ [67]. Similar AMPK pathway activation by RESV has been reported in *in vivo* AD models. In senescence accelerated mouse (SAMP8) model of AD, dietary administration (1 g/kg) of RESV reduced the Aβ burden and Tau hyperphosphorylation via AMPK activation. These results were paralleled with the reduction in cognitive impairment. Moreover, activation of Sirtuin 1 (SIRT1), a class III histone deacetylase enzyme implicated in ROS control [68], was observed in RESV treatment [69]. In APP/PS1 mice model of AD, oral chronic administration of RESV reduced Aβ deposits and increased the protein levels of the mitochondrial complex IV, by activating both SIRT-1 and AMPK pathways [70]. These results suggested that RESV-induced reduction in cognitive impairment in AD models may have resulted via activation of AMPK pathway-mediated Aβ clearance and SIRT1 pathway-mediated prevention of oxidative stress and forkhead transcription factors-induced apoptosis.

Antioxidative and anti-apoptotic effects of RESV have also been investigated in *in vivo* VaD models. Ma *et al.* [71] showed that daily intragastric administration of RESV (25 mg/kg) improved learning and memory ability in a CCH rat model of VaD, by decreasing oxidative stress through MDA reduction, and SOD and glutathione (GSH) upregulation in the hippocampus and cerebral cortex [71]. A recent study reported similar data that in CCH rats, RESV treatment (10 mg/kg) prevented oxidative stress by decreasing lipid peroxidation and restoring the reduced glutathione-S-transferase (GST) level [72]. Sun *et al.* [73] reported that oral doses of RESV (25 mg/kg) attenuated memory impairment in the CCH rat model. This protective effect was supported by the reduction of expression of pro-apoptotic proteins, such as Bax, cleaved caspase-3 and cleaved poly(ADP-ribose) polymerase (PARP). RESV pre-treatment (40 mg/kg) in CCH rats ameliorated spatial learning and memory abilities by restoring the synaptic plasticity, by increasing the activity of protein kinase A (PKA) and by inducing the phosphorylation of the cAMP-responsive element-binding protein (CREB), a critical transcriptional factor involved in the memory process [74]. The summary of molecular mechanisms underlying RESV-induced antioxidative, anti-apoptotic, and anti-amyloidogenic effects discussed above is listed in Table 3. Although all these evidences suggest that RESV possesses a lot of neuroprotective features against dementia, the efficacy of RESV in dementia patients has not yet been demonstrated.

Accordingly, we recommend that clinical trials with dementia patients to evaluate the therapeutic features of RESV may provide more information in the context of the therapeutic implications of RESV in dementia.

### 2.3. Epigallocatechin-3-Gallate: A Flavonoid

**Figure 3:**
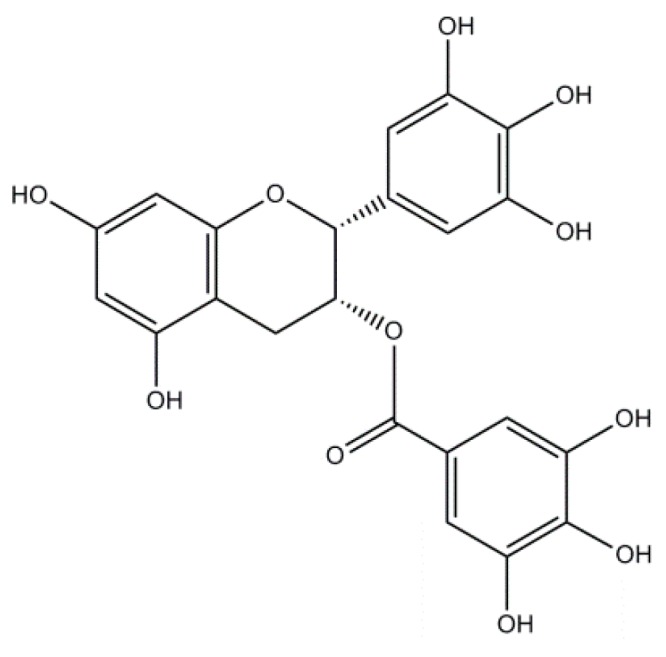
Molecular structure of epigallocatechin-3-gallate.

The flavanol epigallocatechin-3-gallate (EGCG) is the most abundant catechin found in tea, extracted from *Camellia sinensis*, a member of the Theaceae family. EGCG is considered a powerful antioxidant for its direct scavenging properties due to the presence of the trihydroxyl group in the B ring and the gallate moiety esterified at the 3rd position in the C ring [75] (Figure 3). In addition, EGCG possesses the indirect antioxidant ability by activating the nuclear erythroid-2 related factor (Nrf2) and its downstream antioxidant phase II enzymes, including glutathione peroxidase (GPx), glutamate cysteine ligase (GCLC), GST, SOD, NAD(P)H:quinone oxidoreductase 1 (NQO1), and heme oxygenase-1 (HO-1) [76].

Antioxidative and anti-inflammatory effects of EGCG have been investigated in *in vitro* and *in vivo* models associated with AD and dementia. Cheng-Chung *et al.* [77] demonstrated that EGCG treatment of mouse microglia cells (EOC 13.31) suppressed Aβ-induced inflammatory response of microglia by inhibiting the expression of TNF-α, IL-1β, IL-6, and iNOS. Additionally, EGCG protected Neuro2a cells against microglia-mediated neurotoxicity by restoring the levels of Nrf2 and HO-1. In IL-1β/Aβ exposed human astrocytoma cells (U373MG), pre-incubation of EGCG (20 μM) reduced the level of IL-6, IL-8, Vascular Endothelial Growth Factor (VEGF), PGE, and COX2. Activation of NFκB, MAPK and JNK signaling pathways were also inhibited by EGCG [78]. EGCG administration in APP/PS1 mice reduced Aβ level and restored the mitochondrial respiratory rates by decreasing ROS production and by increasing ATP levels in mitochondria derived from the hippocampus, cortex and striatum [79]. Improvement in cognitive impairment and reduction in ROS and AChE activity was observed in icv-STZ rats treated with EGCG (10 mg/kg/day for 4 weeks) [80]. Lee *et al.* [81] showed that EGCG pre-treatment (1.5 mg/kg for three weeks) prevented cognitive impairment in Aβ-treated-mice. In addition, they noticed that EGCG treatment (3 mg/kg for one week) ameliorated the cognitive deficits in AD (PS2-mutant) transgenic mice. *A*β plaques were decreased in both experimental AD mouse models. Interestingly, they found that EGCG inhibited the activation of ERK/NFκB pathway, which resulted in the reduction of Aβ-synthesizing β- and γ-secretases, and increased the activity of non-amyloidogenic α-secretase. Another important study in APP/PS1 mice demonstrated that EGCG may reduce Aβ levels and ameliorate cognitive impairment via two putative mechanisms: (1) neurogenesis induction via nerve growth factor (NGF)-Tropomyosin receptor kinase A (TrkA) pathway activation, which regulates c-Raf/ERK1-2/CREB cascade; (2) apoptosis inhibition, via suppression of pro-apoptotic full length neurotrophin receptor (p75^NTR^)/intracellular domain fragment neurotrophin receptor (p75^ICD^) and reduction of JNK2/cleaved-caspase 3 activity [82]. The summary of molecular mechanisms underlying EGCG-induced antioxidative, anti-inflammatory, and anti-amyloidogenic effects discussed above is listed in Table 4.

Although significant preclinical data from *in vitro* and *in vivo* studies have shown the neuroprotective effects of EGCG, it is important to mention that EGCG, at concentrations of 500 mg/kg body weight and above, has been recognized to be hepatotoxic in mice [83], and sporadic incidents of hepatotoxicity in humans have also been reported [84]. However, clinical trials with EGCG, at daily doses of 800 mg, in AD patients have shown no adverse effects (ClinicalTrials.gov identifier: NCT00951834), and the results of these clinical trials have not been reported yet.

## 3. Isothiocyanates

Isothiocyanates (ITCs), belonging mainly to the family of the Brassicacae (Brussels sprouts, kale, cauliflower and broccoli), are sulfur-containing phytochemicals derived from myrosinase (β-thioglucoside glucohydrolase) hydrolysis of glucosinolates (GLs) [85]. GLs coexist in the same plant, but in separate cells, with the myrosinase enzyme and they are also found within human bowel microflora [86,87]. After mechanical damage of cells, for example, predation/mastication by humans or animals, freeze-thaw injury, or plant pathogens, GLs undergo hydrolysis and release, apart from glucose and sulfate, several biologically active compounds, including ITCs, thiocyanates, and nitriles, depending on the hydrolytic conditions [88,89]. Overall, GLs display a structural homogeneity based on a β-d-glucopyranosyl unit and an *O*-sulfated anomeric (*Z*)-thiohydroximate function connected to a variable side chain depending on the amino acid metabolism of the plant species [90].

The beneficial effects of ITCs consumption have been known since the 1950s, as several studies have reported that regular consumption of Brassicaceae vegetables can contribute to reduce the risk of carcinogenesis and certain chronic diseases, such as cardiovascular diseases and neurodegenerative diseases [91]. In the last three years, ITCs were investigated in the prevention and treatment of cognitive diseases, due to their antioxidant and anti-amyloidogenic features. From a literature search in PubMed, by using the keywords “isothiocyanates and dementia” 23 papers were found, of which 20 papers were focused exclusively on the role of ITCs in AD, highlighting the emerging role of these phytochemicals in the field of dementia.

### 3.1. Sulforaphane

**Figure 4:**
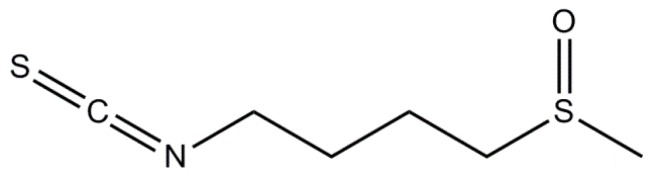
Molecular structure of sulforaphane.

Among ITCs, *R*-sulforaphane (4*R*-1-isothiocyanato-4-(methylsulfinyl)butane; SFN) derived from the enzymatic action of myrosinase on the GL precursor glucoraphanin (GRA; (*R_S_*)-4-methylsulfinylbutyl GL) [92] is the most extensively studied ITC in the course of the past two decades. The configuration of the sulfoxide stereogenic center in the GRA side chain was recently ascertained by NMR to be *R_S_*, a configuration retained in the hydrolysis product *R*-sulforaphane (Figure 4) [93]. In the last decade, SFN has been proven to have neuroprotective activity in both *in vitro* and *in vivo* models of neurodegeneration due to their ability not only to address many targets, but also to modulate different pathways in neuronal cells [94,95,96]. It seems very likely that the beneficial effects of SFN could be mainly ascribed to its peculiar capacity to activate Nrf2/antioxidant response element (ARE) pathway [97]. In addition, many papers published about the biological properties of SFN in experimental models of neurodegeneration have demonstrated that this phytochemical is able to decrease NFκB translocation and consequent production of the main pro-inflammatory cytokines, oxidative species generation and to inhibit neuronal apoptotic death pathway [98,99,100,101].

According to these findings, Lee *et al.* [102] examined the protective effect and the molecular mechanism of SFN against Aβ-induced oxidative and apoptosis. SH-SY5Y cells pre-treated with SFN at different concentrations (1 μM, 2 μM, and 5 μM for 30 min) and exposed for 24 h to Aβ(25-35) showed a direct evidence that SFN protects SH-SY5Y cells from Aβ-induced toxicity through increasing cell viability as well as inhibiting the apoptotic cell death in a dose-dependent manner. Pre-treatment with SFN attenuated also JNK activation via inhibition of its phosphorylation and regulated the ratio of Bax to Bcl-2. Furthermore, it was observed that SFN reduced ROS production by upregulating the expression of antioxidant enzymes, including GCLC, NQO1 and HO-1 through the activation of the Nrf2 pathway. In addition, by using siRNA targeting Nrf2 expression, the same authors further demonstrated that the protective effect of SFN against Aβ-induced apoptotic cell death was mediated via Nrf2 activation [102].

Moreover, several studies performed on neuronal cell lines have shown that the neuroprotective effects of SFN against oxidative stress and Aβ-mediated cytotoxicity could be due in part to the regulation of the proteasome system [103,104,105]. Specifically, Park *et al.* [103] reported that SFN treatment protected Neuro2A and N1E115 murine neuronal cells from Aβ**-**induced oxidative damage and promoted also Aβ clearance, by enhancing the proteasome activity. Furthermore, they observed that the SFN protective effect was abolished by a specific inhibitor of the proteasome, suggesting that SFN protected cells from oxidative damage by increasing the expression of the Nrf2 pathway that in turn enhanced the expression of multiple subunits of the proteasome. Kwak *et al.* [106] reported that SFN protected Neuro2a cells from hydrogen peroxide-mediated cytotoxicity by promoting the proteasome activity via the up-regulation of the proteasome catalytic subunit, 26S. Similar results were obtained in another study performed by Gan *et al.* [104] in which SFN (10 and 7.5 μM) treatment on HeLa and COS-1 cells reduced the level of oxidized proteins and amyloid β by enhancing the proteasome activities through heat shock protein, Hsp27 activation. According to these results, it is likely that the induction of proteasome by SFN may facilitate the clearance of the Aβ aggregates, which leads to the improvement of protein folding in AD.

A study by Brandenburg *et al.* [107] suggested SFN as a good candidate for anti-inflammatory treatment of the central nervous system. Here, the authors demonstrated that SFN administration prevented the anti-inflammatory and pro-apoptotic response induced by LPS stimulation in primary rat microglia and in BV2 microglia cells. In particular, it was demonstrated that SFN reduced the expression of IL-1β, IL-6, and TNF-α and NO production from microglia in a dose-dependent manner through the inhibition of the NF-kB and activator protein-1 (AP-1). SFN was shown also to inhibit LPS-mediated phosphorylation and activation of pro-apoptotic ERK1/2 and JNK. Zhang *et al.* [108] proposed that SFN has potential application in AD therapeutics. SFN oral treatment (25 mg/kg) in mice with AD-like lesions (induced by combined administration of aluminum and d-galactose) reduced the cholinergic neuronal loss by lowering aluminum levels and ameliorated the cognitive impairment. In addition, it was proposed that SFN reduced brain aluminum cargo by accelerating blood aluminum excretion, and also in this model the antioxidative effect of SFN was attributed to its ability to activate the Nrf2 pathway. In a further study, Zhang and coauthors [109], using the same animal model and the same concentration of SFN, investigated the anti-amyloidogenic properties of SFN. They found that SFN administration reduced the numbers of Aβ plaques and caused a significant increase in carbonyl group levels as well as decreased the levels of GPx in the hippocampus and cerebral cortex areas. Since carbonyl formation is an important marker of protein oxidation, results from this study suggested that SFN could exert a protective effect against lipid peroxidation in AD mouse brain by restoring the endogenous antioxidant defenses.

The role of SFN in modulating the cholinergic system has been proven in mouse model of scopolamine-induced memory impairment [110]. In this study, it was demonstrated that oral treatment with SFN (10 or 50 mg/kg) exerted a significant neuroprotective effect on cholinergic deficit and cognitive impairment in mice. Of note, scopolamine is a non-selective muscarinic ACh receptor (mAChR) antagonist that mainly targets M1AChR and M2AChR, thereby impairing learning acquisition and short-term memory in rodents as well as in humans [111], and it was found that SFN improved the cholinergic system reactivity by increasing ACh and choline acetyltransferase (ChAT) levels in the hippocampus and frontal cortex. AChE activity was decreased by SFN. Similar results were obtained in *in vitro* study. SFN (10 or 20 μM) treatment increased ACh level and showed protection in scopolamine-activated primary cortical neurons [110].

In a recent study, Dwivedi *et al.* [112] investigated the role of SFN in rats treated with Okadaic acid (OKA). OKA is an polyether toxins produced by marine microalgae which causes hyperphosphorylation of Tau and development of AD-like symptoms due to its property to inhibit phosphatase activity of PP1 and PP2A phosphatases [113]. The administration of SFN (5 and 10 mg/kg i.p.) in OKA-treated rats ameliorated the cognitive impairment by reducing the release of pro-oxidant species (ROS and nitrite), pro-inflammatory mediators and cytokines (NFκB, TNF-α and IL-10) and blocking neuronal cell death in the hippocampus and cerebral cortex of the OKA-treated rats. Furthermore, they observed that SFN increased Nrf2 expression as well as the expression of the downstream antioxidant enzymes, GCLC and HO-1. In the same study, It was demonstrated that the protective effects of SFN were abolished with Nrf2 siRNA treatment in a rat astrocytoma cell line (C6), suggested the possible Nrf2-dependent activation of cellular antioxidant machinery in SFN-mediated protection against OKA-induced memory loss in rats. Although current evidence indicates that SFN possesses several neuroprotective properties *in vivo* and *in vitro* (as showed in Table 5), clinical trials to test its efficacy in patients suffering from dementia have not yet been investigated.

### 3.2. Moringin

**Figure 5:**
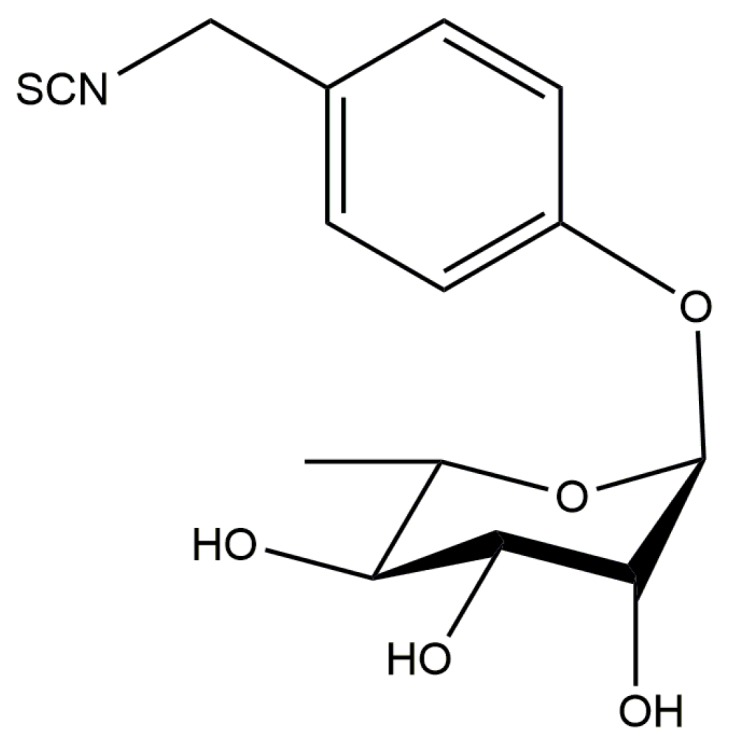
Molecular structure of moringin.

Recently, the attention of researchers has been focused on the study of the glycosylated isothiocyanate moringin (MG) or [4-(α-l-rhamnosyloxy)benzyl isothiocyanate; GMG-ITC], resulting from quantitative myrosinase-induced hydrolysis of glucomoringin (GMG) (4-(α-l-rhamno- pyranosyloxy)benzyl GL), an uncommon member of the arylaliphatic GL class, which is present in fair amounts in vegetables belonging to the family *Moringaceae (*Figure 5) [114]. Growing in many tropical and equatorial areas and commonly known as “horse-radish tree”, *Moringa oleifera* is the most widely distributed species in the genus Moringa [115]. MG has been shown to exert many beneficial activities, including anti-inflammatory as well as antioxidant effects, protecting against neurodegenerative disorders [114,115,116,117,118].

The neuroprotective effect of *M. oleifera* extracts was also investigated in animal models of age-related dementia. AD was induced in rats by bilateral intracerebroventricular administration of the cholinergic neurotoxin ethylcholine ariridinium (AF64A). AF64A-treated rats orally administered with *M. oleifera* leaves extract at doses of 100, 200, and 400 mg/kg for a period of 7 days before and 7 days after the AD induction improved spatial memory and neurodegeneration especially in CA1, CA2, CA3, and dentate gyrus of hippocampus areas. The effects produced by treatment with *M. oleifera* extract may occur partly via the decreased oxidative stress and the enhanced cholinergic function, as proven by reduction of MDA and AChE levels, and increase of SOD, CAT [119].

In addition, *M. oleifera* leaf extract ameliorates memory impairment via nootropic activity and provides notable antioxidants to counteract oxidative stress in rats infused with colchicine (15 µg). Several lines of evidence also suggest that chronic oral treatment with *M. oleifera* at different doses (50, 100, 150, 200, 250, 300 and 350 mg/kg) can alter electrical activity in the brain and the production of monoamines, including norepinephrine, dopamine and serotonin, involved in memory processing, thus ameliorating cognitive functions [120]. It was shown also that this extract increases SOD and CAT enzymatic activity as well as to decrease activity of lipid peroxidase in the cerebral cortex of AD rats by acting as free-radical scavenger [121]. The preclinical studies about the neuroprotective mechanisms of MG are summarized in Table 6.

Although these *in vitro* studies showed the neuroprotective effect of MG in dementia models, further *in vitro* and *in vivo* studies based on different dementia models are required to investigate the efficiency of MG in dementia, which may support to initiate clinical trials in dementia patients with MG treatment.

## 4. Alkaloids

Alkaloids are a class of naturally occurring organic nitrogen-containing compounds extracted from several flowering plants such as the *Papaveraceae, Ranunculaceae, Solanaceae* and *Amaryllidaceae* [122,123,124]. Alkaloids represent a wide and ancient family of compounds with analgesic, antiasthmatic, antiarrhythmic, anticancer, antihypertensive, antipyretics, antibacterial and antihyperglycemic activities. Since the 1960s, the role of alkaloids in the field of dementia has been extensively investigated. The Food and Drug Administration (FDA) approval of the two alkaloid-based drugs, GAL and RIV, for AD treatment in the early 2000s has led to a renewed interest in alkaloids for dementia therapy. In addition, the intrinsic anticholinesterase activity found in alkaloid compounds makes them potential therapeutic agents for dementia.

In this review, we report some of the alkaloids that have shown beneficial effects in the treatment of dementia. Specifically, by a literature search in PubMed we found 61 papers for morphine, 84 and 220 papers for caffeine and nicotine, respectively, 123 papers for huperzine A, and 33 papers for berberine.

### 4.1. Rivastigmine

**Figure 6:**
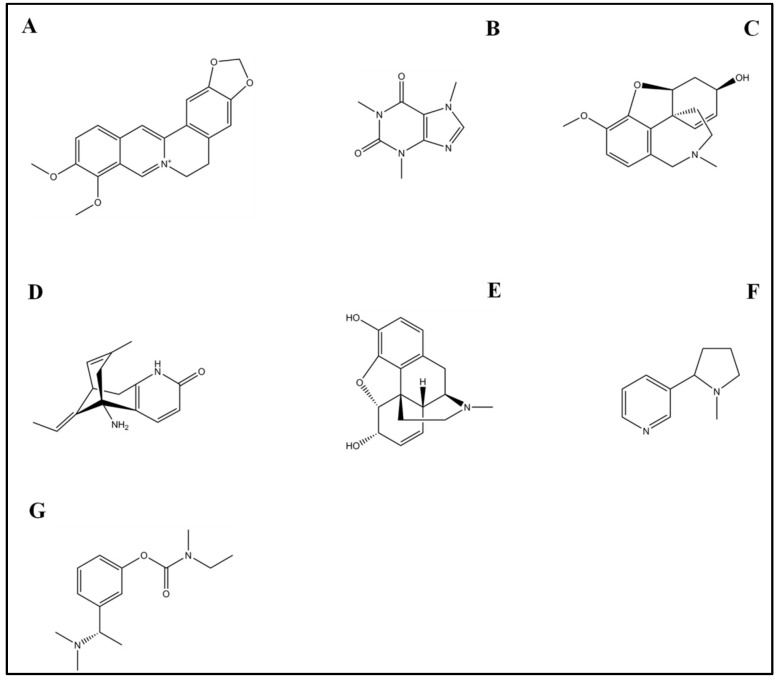
Chemical structures of some alkaloids: (**A**) berberine; (**B**) caffeine; (**C**) galantamine; (**D**) huperzine A; (**E**) morphine; (**F**) nicotine; (**G**) rivastigmine.

RIV (Figure 6G) is a synthetic analog derived from the natural alkaloid physostigmine, isolated from the poisonous seeds of *Physostigma venosum* (Calabar bean) belonging to the *Fabaceae* family [125]. RIV possesses a better therapeutic and safety profile than physostigmine. RIV is a reversible, non-competitive inhibitor of AChE [126]. In 2000 RIV (Exelon^®^) was approved by the FDA as a transdermal patch [127] to treat mild to moderate AD [128], and as of 2014, it has been used for the treatment of AD in more than 90 countries worldwide. Furthermore, from 2006 it has also been used for Parkinson's disease dementia (PDD) [129]. The transdermal patch formulation has shown fewer gastrointestinal side effects than the oral formulation and a higher tolerability rate that permits the administration OF higher doses of RIV in advanced stages of AD [130].

A recent Cochrane review [131] evaluated all controlled, double-blind, randomized clinical trials in which RIV was administered daily orally (6 to 12 mg) as well as transdermally (9.5 mg) in patients with AD for 12 weeks or more. The results of this study showed that RIV ameliorated the cognitive decline function and daily living in patients affected by mild to moderate AD compared with placebo, but did not induce any changes in behavior and in the clinical global assessment.

Of note, the transdermal patch as well as capsules showed comparable efficacy but the transdermal patch manifested fewer side effects than the capsules. Studies in recent years strongly support the efficacy of RIV in AD treatment. The Okayama Rivastigmine Study (ORS) carried out in 2015 [132] analyzed the clinical effects of RIV and donepezil in AD patients at 3, 6, and 12 months. According to this study, it is evident that RIV improved both cognitive and affective functions at 3 and 6 months, showed more benefits compared to donepezil. Ehret *et al.* [133] illustrated in a recent systematic review that AChEIs, including RIV, have consistent but modest effects even in late-phase trials. Additionally, Spalletta *et al.* [134] demonstrated that RIV treatment attenuated the frequency and severity of depressive episodes in patients with mild AD during a 6-month open-label observational study.

In addition to AD and PDD treatment, studies have also suggested a potential therapeutic role of RIV in VaD. In particular, a clinical study evaluated the effect of RIV on the cognitive performance of elderly subjects affected by different subtypes of VaD. After six months of treatment, it was demonstrated that RIV ameliorated the cognitive ability, particularly in patients affected by subcortical ischemic vascular dementia, a VaD subtype characterized by small vessel disease dementia [135]. Furthermore, Birks *et al.* [136], by analyzing different clinical trials, observed that RIV exhibited beneficial effects on vascular cognitive impairment, but it also showed a lot of adverse effects such as vomiting, nausea, diarrhea, anorexia and withdrawals. Although RIV has displayed beneficial effects in patients affected by DLB [137], a recent systematic review reported that RIV has greater risk of adverse events [138]. Moreover, RIV treatment in patients suffering from FTD reduced behavioral impairment and caregiver burden, but failed to prevent the cognitive impairment after 12-month of follow-up.

Overall, RIV is able to slow the cognitive decline in AD patients and some trend of efficacy in the management of behavioral symptoms associated with the disease, while in the other forms of dementia, it exhibits a greater risk of adverse effects.

### 4.2. Galantamine

GAL (Figure 6C) is a synthetic isoquinoline alkaloid, originally extracted in the 1950s from the bulbs and flowers of* Galanthus nivalis* L., belonging to the *Amaryllidaceae* family. GAL has been used in humans for decades as an anesthetic drug and to treat neuropathic pain. To date, after the first approval in Sweden in 2000, GAL (Reminyl^®^, Razadyne^®^) is prescribed in sustained-release capsules to treat mild to moderate AD in European Union as well as in the United States [109,139]. GAL has shown to be a selective, competitive and reversible AChE inhibitor. In particular, it is characterized by two pharmacological mechanisms by which it increases the acetylcholine concentration in the synapses and compensates the decline of cholinergic function in AD patients: (i) the inhibition of acetylcholine esterase and (ii) the allosteric modulation of the nicotinic cholinergic receptor [140].

During the 1990s, clinical studies were focused to investigate the therapeutic potential of GAL in AD patients and to evaluate its safety and efficacy in clinical practice. Particularly, it was demonstrated that GAL administration at 8–32 mg/day resulted in consistent symptomatic improvement of cognitive functions and activities of daily living in patients with mild to moderate AD over 3–6 months [139,141,142]. Also, it was found that GAL (24 mg/day) exerted a sustained effect for 12 months [143]. Richarz *et al.* [144] carried out an open-label trial for three years in order to assess long-term effectiveness of GAL in patients with mild AD. Results showed that after the first year of treatment, GAL improved cognition, behavior, and activities of daily living. Interestingly, after three years the beneficial effect of GAL on cognition was well maintained in AD patients, although a worsening in the general outcomes was recorded. Recently, a clinical study was performed to investigate the influence of cholinesterase inhibitors including GAL, RIV and donezepil on sleep pattern and sleep disturbance in 87 mild to moderate stage dementia patients [145]. In this study, GAL was proved to ameliorate sleep quality compared to treatment with RIV and donepezil, by evaluating the Pittsburgh Sleep Quality Index at the beginning and at the final assessment. Furthermore, GAL has displayed pleiotropic activity in experimental studies such as the ability to inhibit Aβ aggregation and cytotoxicity *in vitro* [146] and to prevent Aβ-induced oxidative stress [147], due to its scavenging properties [148,149].

Only a limited number of clinical studies have evaluated the therapeutic relevance of GAL in the other forms of dementia. In a randomized double-blind trial, Birks *et al.* [150] found that GAL treatment (at the dose of 16–24 mg/day) in VaD patients ameliorated the cognitive impairment and the global assessment, and showed good safety and tolerability [151]. However, gastrointestinal side-effects were observed in these patients. In another clinical study, Edwards *et al.* [152] tested GAL efficacy and safety in a cohort of 50 patients affected by DLB and found that GAL attenuated the neuropsychiatric symptoms associated with the disease such as hallucinations. Moreover, GAL-induced side effects were mild and transient. According to these findings, O'Brien *et al.* [153] affirmed that AChEIs, including GAL, can improve cognitive performance in DLB patients and suggested the application of AChEIs especially for the treatment of neuropsychiatric symptoms associated with DLB. Another open clinical trial evaluated the effect of GAL treatment for a period of 8 weeks in a cohort of patients affected by the two most common varieties of FTD: the behavioral variety FTD and the primary progressive aphasia. Results from this trial reported that GAL showed a trend of efficacy only in patients affected by the aphasic variety of FTD according to the clinical global impressions scale [154].

A recent meta-analysis [155] evaluated whether treatment with AChEIs could provide cognitive benefits in VaD patients. Here, it was found that patients treated with donepezil as well as with GAL showed relevant improvement in Alzheimer's Disease Assessment Scale-cognitive subscale (ADAS-cog) compared to the placebo group, but not in the Mini Mental State Examination (MMSE). Conversely, RIV treatment did not show any benefit on AD [155].

Overall, GAL has demonstrated to slow cognitive decline in AD patients and thus to be useful in the management of some behavioral symptoms. Furthermore, it has shown some efficacy in VaD, DLB and FTD patients. However, we assume that the achieved results in these forms of dementia need further validation.

### 4.3. Morphine

Morphine (MOR, Figure 6E) is a benzylisoquinoline alkaloid first isolated from *Papaver somniferum* about 200 years ago. MOR is considered an opiod compound as it targets the opioid receptors. Since the 1950s MOR is recognized as one of the leading analgesics for alleviating acute and chronic pain and it has been also administered in palliative care in the terminal stages of cancer [156]. MOR is also considered a narcotic drug, characterized by important side effects such as heavy sedation and physical dependence, and as such it was added to the list of narcotic drugs.

In the last years, literature data has reported that MOR possesses anti-amyloidogenic properties in experimental models of AD. MOR treatment in rat primary neuronal cultures as well as in APP/PS1 mice was shown to protect against Aβ toxicity by promoting the estradiol release from neurons and by up-regulating the Heat shock protein-70 (Hsp70), which in turn restores the proteasome activity impaired by Aβ [157]. In addition, Wang *et al.* [158] showed that MOR pre-treatment attenuated Aβ oligomers-induced neurotoxicity in primary cultured cortical neurons in a dose-dependent manner. This effect was shown to be dependent on activation of μ-opioid receptor and was mediated by reversal of Aβ oligomers-induced downregulation of mTOR signaling. The role of mTOR pathway has been widely investigated in the pathogenesis of AD. Indeed, mTOR signaling is involved in modulating long-lasting synaptic plasticity [159] and the consolidation of long-term learning and memory [160] processes, which are dramatically impaired during AD. These studies suggest opioid receptors as potential therapeutic targets for AD. To date, there are no relevant data reported in the literature on the use of MOR in the treatment of VaD or other forms of dementia.

MOR has also been evaluated in the management of dementia-related symptoms. In a randomized clinical trial in patients with moderate to severe AD, Husebo *et al.* [161] demonstrated that pain treatment with MOR seems to reduce agitation behaviors. Furthermore, the same group, in a previous clinical study, has observed that MOR administration in these patients could ameliorate mood symptoms including depression [162]. Although the preclinical results (summarized in Table 7) indicated that MOR could be protective against Aβ toxicity and clinical studies suggested that MOR may help to manage some AD symptoms, its sedative side effects could be a limiting factor for its potential application in the treatment of dementia.

### 4.4. Caffeine

Caffeine (CAF, 1,3,7-trimethylxanthine, Figure 6B) is a purine alkaloid isolated from coffee plants (*C. arabica* L.), present in high concentrations in beverages, including coffee, tea, soft drinks and chocolate. It is considered a non-selective antagonist of the adenosine A2A receptor [163]. Literature data regarding the role of CAF in several human diseases are still controversial. However, epidemiological and observational studies have suggested that habitual consumption of CAF can be associated with a decreased risk to develop Parkinson’s disease [164,165,166], which encouraged other studies in the field of the neurological disorders.

Indeed, preclinical studies have proposed that CAF intake can prevent memory decline during aging and can reduce the risk to develop dementia and particularly AD [167,168,169]. In line with these findings, Laurent *et al.* [170] demonstrated that chronic CAF administration (10 months) through drinking water (0.3 g/L) in the THY-Tau22 transgenic mousemodel of progressive AD improved spatial memory performance in the Morris Water Maze test. In addition, CAF treatment significantly reduced hippocampal Tau phosphorylation and the respective proteolytic Tau fragments in THY-Tau22 mice, and these effects were paralleled by down-regulation of inflammatory mediators (TNF-α, GFAP and MAPK) and oxidative stress markers (Nrf2 and manganese-dependent superoxide dismutase (MnSOD)) [170]. In line with these findings, Arendash *et al.* [171] demonstrated that daily CAF administration (1.5 mg/mouse, corresponding to five cups of coffee/day in humans) for six weeks in drinking water decreased hippocampal Aβ levels by reducing the expression of PS1 and BACE-1in young Tg APP_swe_ mice. In the same study, the effect of CAF to reduce Aβ production was confirmed in N2a/APP_swe_ cells, where concentration-dependent reduction in both Aβ_1–40_ and Aβ_1–42_ was observed [171]. In another study, the same authors, using old TgAPP_swe_ mice (aged 18–19 months), found similar results, demonstrated that CAF administration reduced Aβ burden and the memory impairment [172]. Han *et al.* [173] showed that low (0.75 mg/day) and high (1.5 mg/day) doses of CAF administered for eight weeks in APP/PS1 double transgenic mice ameliorated the spatial learning and memory abilities and increased the hippocampal expression of BDNF and its receptor, the tropomyosin receptor kinase B (TrkB), in a dose dependent manner. The role of BDNF and TrkB in the pathophysiology and cognitive deficits of AD has been well reported in previous studies [174] and it seems that the protective role of CAF against memory impairment in AD might be resulted from the activation of BDNF/TrkB signaling. Furthermore, in a rabbit model of sporadic AD induced by cholesterol-enriched diet, CAF administration (0.5 and 30 mg/day for 12 weeks in the drinking water) restored the increased levels of Aβ and phosphorylated Tau, and decreased the oxidative stress levels induced by cholesterol [175]. The summary of molecular mechanisms of CAF described earlier is listed in Table 8.

The beneficial effects of CAF in AD progression and prevention have been evaluated in several clinical studies. In Maia and de Mendonça study [167] the association between CAF intake and AD risk was investigated by comparing AD patients, who had an average daily caffeine intake of 74 mg during the 20 years that preceded the diagnosis of AD, with the healthy controls who had an average daily CAF intake of 199 mg during the corresponding 20 years of their lifetimes. By logistic regression analysis it was found that the CAF intake during this period was inversely associated with AD. Similarly, another study reported that coffee drinkers (3–5 cups per day) at midlife had lower risk to develop dementia compared with those drinking no or only little coffee [176]. In another case-control study with two separated cohorts of elderly (65–88 years old), it was observed that high plasma CAF levels were associated with a reduced risk to develop dementia [169]. However, controversial results have also been documented in clinical trials. A latest meta-analysis of observational epidemiological studies found that there was no correlation associated between CAF intake and the risk of cognitive disorders [177].

Similarly, Gelber and colleagues [178] proved a lack of association between coffee intake and development or progression of cognitive impairment, overall dementia, AD, VaD, or moderate/high levels of the individual neuropathologic lesion types. Consequently, further clinical trials with longer follow-up periods are needed to investigate the relationship between CAF and AD development.

### 4.5. Nicotine

Nicotine (NIC, Figure 6F) is a pyrrolidine alkaloid isolated from tobacco (*Nicotiana tabacum* L.) leaf and it is also the main psychoactive component of tobacco smoke. Although smoking is associated with negative health effects, the pure form of NIC has been investigated by researchers as potential therapeutic agent in AD [179] since NIC is an allosteric modulator of the ACh nicotininc receptors (nAChRs). Indeed, a great amount of studies has reported that the activation of brain nAChRs can potentiate the cholinergic system, representing an important therapeutic target in AD [180,181,182]. According to these findings, NIC has shown a neuroprotective effect against Aβ toxicity and neuroinflammation [183]. Structural and *in vitro* studies demonstrated that NIC was able to break down preformed Aβ fibrils due to the ability of its *N*-methylpyrrolidine moieties to bind with the Aβ histidine residues, which exerted a neuroprotective effects [182,184].

*In vivo* chronic administration of NIC (2 mg/kg for 6 weeks) in AD rat model reduced BACE-1 expression and Aβ levels, attenuated the Aβ-induced memory and learning impairment and furthermore, prevented the decreased expression of the nicotinic receptors α_7_- and α_4_-nAChR induced by Aβ [185]. Moreover, in transgenic mice (aged 12 months) expressing neuron-specific enolase (NSE)-controlled APP_swe_, low, middle, and high doses treatment of NIC for 6 months displayed an improvement in memory and increased the expression of nAchRα7 receptors [186]. Likewise, in another study, it has been demonstrated that male Wistar rats subjected to intermittent and repeated exposure of NIC (0.35 mg/kg every 12 h for 14 days) improved memory performance and increased the expression of choline acetyltransferase (ChAT), vesicular ACh transporter (VAChT) and NGF receptor, TrkA [187]. Conversely, another study found that chronic NIC-treated-water supplementation in the transgenic AD mouse model (3xTg) increased the levels of nicotinic receptors that in turn increased Tau aggregation and phosphorylation state, which eventually exacerbated Tau pathology [188]. Similarly, a study of Deng *et al.* [189] reported that NIC did not improve cognitive impairment in rats Aβ-injected, and increased Aβ-induced Tau phosphorylation. Until now, the effects of NIC in VaD and in FTD have not been investigated. Ono *et al.* study [190] demonstrated that NIC inhibited *in vitro* α-Syn aggregation in a dose-dependent manner, suggested a protective role of NIC in DLB. The molecular mechanisms of NIC described earlier are summarized in Table 9.

The therapeutic value of NIC has been investigated in clinical trials. A double-blind, cross-over study reported that NIC transdermal patches, worn for 16 h a day at the following doses: 5 mg/day during week 1, 10 mg/day during week 2 and week 3 and 5 mg/day during week 4, improved only attentional performance in AD patients but not motor and memory abilities [191]. In addition, Newhouse *et al.* [192] demonstrated in a preliminary study that NIC therapy for six months through transdermal patches (15 mg/day) improved cognitive test performance in patients with mild cognitive impairment but did not ameliorate the clinical global impression. However, further clinical studies are required to have more data in order to assess the therapeutic relevance.

### 4.6. Huperzine A

Huperzine A (HupA, Figure 6D) is an alkaloid compound extracted from the Chinese herb *Huperzia serrata*. The beneficial effects of HupA were discovered centuries ago, when HupA was administered to treat different diseases such as fever, rheumatism, schizophrenia, contusions and myastenia gravis [193].

A review by Ma *et al.* [193], by collecting the data obtained from different clinical trials performed in China in AD patients (around 100,000), reported that HupA significantly improved memory deficits. The convincing results obtained about the efficacy of HupA have led to its approval in China for AD treatment in 1994 [194].

Furthermore, in the last decade, many *in vitro* and *in vivo* studies have elucidated the neuroprotective effects of HupA. Tang *et al.* [195] showed that HupA pre-treatment (10 µM) protected neuroblastoma cells SHSY5Y from hydrogen peroxide (H_2_O_2_)-induced toxicity in part by up-regulating the expression of NGF and its receptors P75^NTR^ and TrkA and in part by activating the ERK/MAPK signal pathway, suppressing H_2_O_2_-induced apoptosis. Antioxidative property of HupA has been investigated in different cell lines exposed to Aβ-induced toxicity. HupA treatment increased the level of antioxidant enzymes, such as GPx and CAT, enhanced the level of ATP to improve the mitochondrial energy metabolism, and reduced ROS accumulation, cleaved-caspase-3 expression and nuclei fragmentation induced by Aβ toxicity [196,197,198]. HupA has shown a greater penetration ability to cross BBB [199] and higher AChE inhibitory effects compared to that of tacrine, donezepil and RIV drugs [200] in *in vivo* studies. Additionally, the neurogenesis effect of HupA has been observed in the subgranular zone of the hippocampus in adult mice, via MAPK/ERK signaling pathway activation [201]. Anti-amyloidogenic effects of HupA have been demonstrated in rats subjected to Aβ icv-infusion. Daily i.p. administration of HupA (0.1–0.2 mg/kg for 12 consecutive days) significantly ameliorated learning deficits. Furthermore, HupA reduced apoptosis by up-regulating the anti-apoptotic Bcl-2 and downregulating the expression of pro-apoptotic Bax and p53 [202]. Another study showed significant reduction of Aβ accumulation in HupA treated rats exposed to Aβ. In this study, the authors suggested that HupA might increase the activity of PKC, which stimulated the non-amyloidogenic pathway of APP formation. Similar PKC activation was noticed in mutant APP_swe_ (HEK293_swe_) expressing HEK293 cells treated with HupA [203].

A summary of molecular mechanisms underlying HupA-induced antioxidative and anti-amyloidogenic effects discussed above is listed in Table 10. It has been demonstrated that, in dementia patients, HupA showed a selective inhibitory effect against AChE and increased the ACh level in the brain. Cognitive impairment was ameliorated by HupA treatment. In addition, HupA reduced the glutamate excitotoxicity by antagonising NMDA receptors [204]. As regard VaD, HupA was found to improve the cognitive function in a randomized double-blind clinical trial, performed on a cohort of patients with mild to moderate VaD [205], and confirmed also by a meta-analysis study [194]. However, a systematic Cochrane review reported controversial data about the efficacy of HupA in VaD patients [206].

Overall, HupA may represent one of the most interesting alkaloid candidates in dementia, since several clinical studies have demonstrated its efficacy in AD treatment and preclinical studies have highlited its neuroprotective features. Nevertheless, controversial evidence from the literature suggests that large scale clinical trials are required to validate the efficacy of HupA in dementia.

### 4.7. Berberine

Berberine (BER, Figure 6A) is a natural isoquinoline alkaloid extracted from *Coptis chinensis* and other plants, which displays a lot of pharmacological benefits such as antioxidative [207], anti-inflammatory [208], neuroprotective, antitumor [209] and antimalarial [210]. Like HupA, BER has been used for centuries in Chinese herbal medicine to cure different kind of infections [211]. Since the 1970s BER has been widely investigated by researchers for its anti-tumor properties [212].

Recently, the attention of researchers has grown considerably with regard to the role of BER in dementia. It has been reported that BER acts on the central nervous system by crossing the BBB [213,214], where it increases the cholinergic transmission by inhibiting AchE and butyryl-cholinesterase (BChE) [215]. The role of BER in cholinergic transmission has been greatly explored in *in vitro* and *in vivo* models of AD [216,217]. BER could reduce Aβ production by enhancing the non-amyloidogenic pathway. Asai *et al.* [218] showed that BER treatment in APP_swe_ variant expressing human neuroglioma H4 cells significantly reduced the level of Aβ accumulation, in part by stimulating the α-secretase activity and in part by inhibiting the β-secretase, which resulted in a shift of APP processing towards non-amyloidogenic pathway. A similar reduction of β-secretase and Aβ synthesis was observed in HEK293-APP_swe_ cells treated with BER. This study suggested that BER-mediated ERK1/2 pathway activation may attenuate β-secretase and Aβ synthesis [219]. Moreover, BER administration in N2a/APP_swe_ cells inhibited Aβ generation and Tau hyperphosphorylation by modulating the Akt/GSK3β signaling pathway. Interestingly, BER administration for 4 months (25 mg/day) in AD transgenic mouse model (TgCRND8) reduced the learning deficits as well as ameliorated the long-term spatial memory impairment [43].

Antioxidative and anti-inflammatory effects of BER have also been investigated in *in vitro* model of AD. In normal rat primary astrocytes, BER treatment (10 μM) increased the HO-1 expression in a dose-dependent manner, by activating the PI3-kinase/Akt pathway [220]. In Aβ exposed murine microglia BV2 cells, BER treatment (5 μM) decreased the release of IL-6, monocyte chemoattractant protein-1 (MCP-1), iNOS, and COX-2, via inhibiting MAPK and NFκB signaling [221]. However, it is important to mention that some *in vitro* and *in vivo* studies have demonstrated the neurotoxic effects BER in PD model. Kwon *et al.* [222] observed that BER administration aggravated the 6-hydroxydopamine (6-OHDA)-induced cytotoxicity in PC12 cells and promoted *in vivo* degeneration of dopaminergic neuronal cells in the substantia nigra of OHDA-lesioned rats. In addition, BER treatment decreased the levels of dopamine, norepinephrine, 3,4-dihydroxy- phenylacetic acid (DOPAC) and homovanillic acid (HVA) in the striatum. The summary of molecular mechanisms underlying BER-induced antioxidative and anti-amyloidogenic effects discussed above is listed in Table 11.

Many clinical trials have reported the therapeutic effects of BER in patients with various diseases, such as type 2 diabetes [223] non-alcoholic fatty liver disease [224] and diarrhea-predominant irritable bowel syndrome [225]. However, its neuroprotective effects in dementia patients has yet to be demonstrated. Combined neuroprotective and neurotoxic effects obtained from preclinical studies with different models described earlier suggest the requirement of further evaluation of protective and adverse effects of BER in dementia models, which may assist to commence BER-based clinical trials in dementia patients.

## 5. Phytocannabinoids

Phytocannabinoids (pCBs) are lipid-soluble phytochemicals present in the plant *Cannabis sativa* L., used for a thousand years for both recreational and medicinal purposes [226]. pCBs are terpenophenol compounds, produced by the enzymatic condensation of a terpenic moiety (geranyl diphosphate) with a phenolic group (mainly olivetolic or divarinic acid) [227].

**Figure 7:**
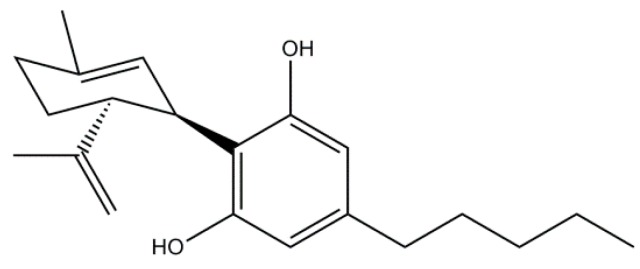
Molecular structure of cannabidiol.

By a literature search in PubMed, we found that the application of pCBs in the treatment of human diseases has been developed in an exponential manner in the last decade. Particularly, by using the keywords “cannabinoids and dementia” 105 papers were found, of which 59 have been published from 2010 to 2016. Interestingly, the recent interest of researchers for cannabinoids is focused not only on their role in alleviating AD-related symptoms but also as potential neuroprotective compounds. Indeed, many Galenic formulations of two important pCBs, namely cannabidiol (CBD, Figure 7) and delta-9 tetrahydrocannabinol (Δ^9^-THC) in different percentages are available in the market (Bedrocan^®^***,*** Bedrobinol^®^*,* Bediol^®^, Bedrolite^®^ and Bedica^®^).

Among pCBs, Δ^9^-THC is the most widely investigated one, but the predominant psychotropic activity strongly limiting its therapeutic use as an isolated agent. Consequently, the therapeutic efficiency of CBD, a non-psychoactive compound, has been studied in central nervous system diseases. Most of the evidence supporting the potential therapeutic utility of CBD in AD have been obtained by using *in vitro* and *in vivo* models of dementia variety of AD-related changes.

### Cannabidiol

An *in vitro* study demonstrated that treatment with CBD (10^−7^ to 10^−5^ M) inhibited hyper-phosphorylation of Tau protein in Aβ-stimulated PC12 neuronal cells via the reduction of the phosphorylated active form of GSK-3β, one of the known Tau kinases, that leads to rescue Wnt/β-catenin pathway and subsequent reduction of neuronal cell loss [228]. In a further study, the same authors showed that CBD treatment (10^−6^ to 10^−4^ M) reduced the expression of iNOS in Aβ-stimulated PC12 cells, by inhibiting the phosphorylation of MAPK, limiting the transcription of pro-inflammatory downstream genes and preventing the translocation of NFκB into the nucleus [229].

The evidence that CBD exerts a combination of neuroprotective, antioxidative and anti-apoptotic effects against Aβ-peptide toxicity was provided by Iuvone *et al.* study [230] which showed that CBD (10^−7^–10^−4^ M) treatment in PC12 cells exposed to Aβ prevented ROS production, lipid peroxidation, and reduced apoptosis by down-regulating caspase 3 expression, DNA fragmentation and intracellular calcium concentration. Moreover, in similar conditions CBD reduced the levels of NO and iNOS, thus confirming its antioxidative properties [231]. In addition, both CBD and Δ^9^-THC prevented the cell death in glutamate induced toxicity in rat cortical neurons, and this neuroprotection was mediated by NMDA, α-amino-3-hydroxy-5-methyl-4-isoxazolepropionic acid (AMPA) and kainate receptors [232]. Besides, CBD protected neuron cultures against hydroperoxide toxicity and showed about 30%–50% more efficacy against oxidative stress compared to other antioxidants such as α-tocopherol or ascorbate [232].

Scuderi *et al.* [233] investigated CBD as a possible modulating compound of APP in transfected human neuroblastoma SHSY5Y^APP+^ cells. They demonstrated that CBD treatment (10^−9^ to 10^−6^ M) induced the ubiquitination of APP protein, leading to a significant decrease in APP full length protein levels in SHSY5Y^APP+^ with the consequent decrease in Aβ production. Additionally, CBD promoted an increased survival of SHSY5Y^APP+^ cells, reduced apoptotic rate and increased the survival over long periods in culture. In line with these findings, the anti amyloidogenic, anti-inflammatory and antioxidant properties of CBD were also demonstrated in *in vivo* studies, supporting the consideration of a cannabis-based medicine as a potential therapy in AD. The role of PPARγ in the mediating anti-inflammatory and neuroprotective effects of CBD was examined in rat AD model. Specifically, it was demonstrated that administration of CBD (10 mg/kg, i.p.) in Aβ-injected rats, antagonized the Aβ-mediated release of pro-inflammatory molecules and cytokines (NO, IL-1β and TNF-α). However, by using a PPARγ antagonist, it was observed that CBD effect was completely suppressed, suggested that CBD activities are regulated by PPARγ [234].

In mice inoculated with human Aβ (1-42) peptide into the right dorsal hippocampus, CBD treatment (2.5 or 10 mg/kg^−1^, i.p.) attenuated Aβ plaques formation, modulated iNOS expression, and decreased MAPK and NFκB levels. In this experiment, CBD suppressed the production of proinflammatory molecules, including IL-1β and NO, thus limiting the propagation of neuro-inflammation and oxidative stress, in a dose-dependent manner [235]. Martín-Moreno *et al.* [236] showed that subchronic and systemic administration of CBD (20 mg/kg for 3 weeks) as well as synthetic cannabinoid WIN 55,212-2 in Aβ-injected mice improved learning behavior. Furthermore, they demonstrated that CBD treatment modulate the microglial cell function and cytokine expression.

To date, the only commercially available preparation containing cannabinoids is Sativex^®^ (GW Pharma, Ltd., Salisbury, Wiltshire, UK), an oral spray consists approximately 1:1 mixture of Δ^9^-THC:CBD in an aromatized water-ethanol solution, approved for the treatment of spasticity and pain in some forms of multiple sclerosis (MS) [237]. In a recent work, Aso and coworkers [238] tested the therapeutic effects of the combination of Δ^9^-THC + CBD (0.75 mg/kg each) in a APP/PS1 transgenic mouse which mimics the most common features of the disease, including cognitive impairment and several pathological alterations, such as Aβ deposition, dystrophic neurites, synaptic failure, mitochondrial dysfunction, and oxidative stress damage. They demonstrated that administration of the mixture of these two compounds in the early stage of the pathology reduced the expression of several cytokines and pro-inflammatory mediators in APP/PS1 transgenic mice, preserved memory and reduced learning impairment [238]. In addition, a considerable decrease in soluble Aβ (1–42) peptide levels and a change in plaques composition were observed in Δ^9^-THC + CBD-treated APP/PS1 transgenic mice, due to a reduced microgliosis and expression of several cytokines and related molecules of neuro-inflammation [238]. In this study, the authors suggested that the combination of Δ^9^-THC + CBD exhibits a better beneficial effect than each *Cannabis* component alone.

Moreover, the effects of pCBs on the regulation of cerebral blood flow may contribute to their potential benefits in AD. Several studies have proved that cannabinoids may cause vasodilation of brain blood vessels and consequently increase cerebral blood flow [239,240]. As decreased cerebral blood flow in AD contributes to the reduction of oxygen and nutrients in brain [8], it is possible that treatment with cannabinoids could improve cerebral perfusion. In this context, CBD was shown to reduce the infarct volume in animal models of focal or global cerebral ischemia, and since VaD is a consequence of brain ischemia, it was suggested that CBD may prevent VaD [241]. In mouse model of focal ischemia with middle cerebral artery (MCA) occlusion used for studying VaD, CBD treatment (3 mg/kg) before and 3 h after MCA occlusion reduced the infarct volume and increased cerebral blood flow. Furthermore, it was demonstrated that the neuroprotective effect of CBD was inhibited by a serotonin 5-hydroxytriptamine1A (5-HT1A) receptor antagonist (WAY100135), suggested that CBD prevented cerebral infarction though a serotonergic receptor-dependent mechanism [242]. The summary of molecular mechanisms underlying CBD discussed above is listed in Table 12.

Currently, there are few data regarding clinical effects of pCBs on human AD. A single, open-label, non-placebo controlled study performed in AD patients reported that dronabinol, derived from Δ^9^-THC, has a beneficial role in reducing anorexia and improving behavior, like nocturnal motor activity and agitation [243]. Similarly, one clinical trial, including 15 patients suffering from AD showed a decreased severity of altered behavior and an increase in the body weight in AD patients after 6 weeks of treatment with dronabinol. Adverse effects associated with this treatment were limited to euphoria, somnolence and tiredness, but these effects did not warrant discontinuation of therapy [244]. Moreover, a systematic Cochrane review identified only one study that meets the criteria to assess the efficacy of cannabinoids to treat dementia [245]. However, since the data are insufficient, the effectiveness of cannabinoids in the improvement of behavior and other parameters of dementia patients are still unclear. Therefore, more controlled trials are needed to assess the effectiveness of pCBs in the treatment of dementia and even more for VaD and other dementia since there are still no clinical studies in these forms.

## 6. Conclusions

New drug candidates acting on multiple molecular targets for the treatment of dementia are urgently required. In this review, we aimed at elucidating the pleiotropic effects of some phytochemicals, belonging to the polyphenol, isothiocyanate, alkaloid and cannabinoid families, and their ability to target in parallel several pathological pathways involved in dementia.

Polyphenols have displayed antioxidative, anti-amyloidogenic and anti-inflammatory properties in preclinical studies, representing interesting candidates in the prevention and treatment of dementia. Nevertheless, clinical trials for therapeutic assessment of polyphenols in dementia patients have not shown encouraging data. Isothiocyanates have exhibited antioxidative properties in AD models, suggesting a potential role in dementia treatment. However, clinical studies to assess their efficacy are still missing.

Alkaloids have displayed a trend of efficacy in the management of behavioral symptoms associated with AD, but in parallel they have also shown side effects, such as toxicity or addiction properties. To date, among alkaloids, HupA is likely to be the most interesting candidate for dementia treatment.

CBD is one of the most promising cannabinoid family members, lacking psycoactive properties and characterized by antioxidative and anti-inflammatory features. However, clinical studies have not shown promising results.

Therefore, our opinion is that phytochemicals could represent an important resource in the development of new medications or as starting point to develop new synthetic analogs or alternatively, they can be associated to conventional therapies for dementia. However, an exhaustive amount of clinical evidence are missing or controversial, and new designed clinical trials are required to better understand their therapeutic or preventive potential in dementia.

## Figures and Tables

**Table 1:** Polyphenols are classified into two main groups: non-flavonoids and flavonoids. Non-flavonoids include phenolic acids, stilbenes, and lignans. Flavonoids are distinct in six subgroups: flavones, flavonols, flavanols, flavanones, isoflavones, and anthocyanins.

Subclass	Polyphenols	Source
	*Non-Flavonoids*	
	Phytochemical	
Stilbenes	resveratrol	grapeskin, red wine, blueberries and blackberries
Lignans	secoisolariciresinol	linseed, cereals and grain
	***Flavonoids***	
Flavones	apigenin, luteolin	parsley and celery
Flavonols	kaempferol, quercetin	onions, leeks and broccoli
Flavanols	catechin, epicatechin, epigallocatechin and epigallocatechin gallate	green tea, red wine and chocolate
Flavanones	hesperetin, naringenin	citrus fruits and tomatoes
Isoflavones	daidzein, genistein, glycetin	soy and soy products
Anthocyanins	pelargonidin, cyanidin, malvidin	red wine and berry fruits

**Table 2:** Preclinical studies of curcumin-mediated neuroprotective effects.

Model	CUR-Mediated Protective Effects	Proposed Mechanisms Involved	Up/Down	References
		***In vitro***		
LPS-stimulated rat BV2 microglia	antioxidative, anti-inflammatory	iNOS, NO, COX-2, PGE2, IL-1β, IL-6, TNF-α	↓	[37]
Aβ-induced murine primary microglia	anti-inflammatory, anti-amyloidogenic	IL-1β, IL-6, TNF-α, MAPK, ERK1/2	↓	[38]
Aβ-induced rat PC12 cells	anti-amyloidogenic	intracellular calcium, Tau hyperphosphorylation	↓	[42]
Mutant APP_swe_ over expression in SH-SY5Y	anti-amyloidogenic	GSK3β activity, APP and Tau hyperphosphorylation	↓	[43]
Mutant APP_swe_ over expression in Neuro2A	anti-amyloidogenic	PS1, BACE-1, Aβ plaques	↓	[37]
		***In vivo***		
Tg2576 mice expressing mutant APP	anti-inflammatory, anti-amyloidogenic	IL-1β, GFAP, amyloid plaques	↓	[39]
Icv-STZ mice model for AD	anti-inflammatory, antioxidative	AChE, oxidative stress, memory deficits PPARγ receptor activation	↓ ↑	[40]
APP/PS1 double transgenic AD mice	anti-amyloidogenic	Aβ deposits, cognitive deficit	↓	[38]
APP/PS1 double transgenic AD mice	anti-amyloidogenic	PI3K/Akt/mTOR pathway	↓	[46]
APP/PS1 double transgenic AD mice	anti-amyloidogenic	insulin-degrading enzymes and neprilysin	↑	[47]
CCH rats	anti-cholesterol	ATP-binding cassette transporter and Apolipoprotein A1	↑	[50]

**Table 3:** Preclinical studies of resveratrol-mediated neuroprotective effects.

Model	RESV-Mediated Protective Effects	Proposed Mechanisms Involved	Up/Down	References
	***In vitro***			
Aβ-induced rat C6 glioma cells	anti-inflammatory	iNOS, NO, COX-2, PGE2	↓	[62]
Aβ-induced rat PC12 cells	anti-apoptotic anti-inflammatory	ROS, Bax, JNK, NFκB	↓	[63]
Aβ-induced rat hippocampal cells	anti-apoptotic	PKCphosphorylation	↑	[64]
Mutant APP_swe_ over expression in Neuro 2A and in HEK293 cells	anti-amyloidogenic	AMPK	↑	[66]
	***In vivo***			
Healthy rats	antioxidative	SOD, CAT MDA	↑ ↓	[61]
SAMP8 mice	anti-amyloidogenic antioxidative	AMPK, SIRT-1	↑	[68]
APP/PS1 double transgenic AD mice	anti-amyloidogenic antioxidative	AMPK, SIRT-1	↑	[69]
CCH rats	antioxidative	MDA GSH, SOD, GST	↓ ↑	[71] [72]
CCH rats	anti-apoptotic	Bax, PARP	↓	[73]
CCH rats	spatial learning and memory improvement	PKA, CREB phosphorylation	↑	[74]

**Table 4:** Preclinical studies of the epigallocatechin 3-Gallate-mediated neuroprotective effects.

Model	EGCG-Mediated Protective Effects	Proposed Mechanisms Involved	Up/Down	References
	***In vitro***			
EOC 13.31	anti-inflammatory	TNF-α, IL-1β, IL-6, iNOS.	↓	[77]
Neuro2a	antioxidative	Nrf2, HO-1	↑	[77]
IL-1β/Aβ exposed U373MG cells	anti-inflammatory	IL-6, IL-8, VEGF, PGE, COX2. NFκB, MAPK, JNK	↓	[78]
	***In vivo***			
APP/PS1 double transgenic AD mice	antioxidative anti-amyloidogenic	ROS ATP	↓ ↑	[79]
icv-STZ rats	anti-amyloidogenic anti-oxidative	ROS, AChE	↓	[80]
AD (PS2-mutant) transgenic mice; Aβ-treated mice	anti-amyloidogenic	ERK/NFκB, γ-secretases, β-secretases	↓	[81]
APP/PS1 double transgenic AD mice	neurogenesis anti-amyloidogenic anti-apoptotic	NGF, TrKa p75^NTR,^ JNK/cleaved-caspase 3	↑ ↓	[82]

**Table 5:** Preclinical studies of sulforaphane-mediated neuroprotective effects.

Model	SFN-Mediated Protective Effects	Proposed Mechanisms Involved	Up/Down	References
	***In vitro***			
Aβ-exposed SHSY5Y cells	anti-apoptotic antioxidative	JNK Nrf2	↓ ↑	[102]
Neuro 2A cells N1E115 cells	anti-amyloidogenic antioxidative	Nrf2	↑	[103]
Hela and COS-1 cells	antioxidative anti-amyloidogenic	Hsp27	↑	[104]
BV2 microglia cells	anti-inflammatory anti-apoptotic	NFκB, ERK1/2, JNK	↓	[107]
	***In vivo***			
Scopolamine-infused mice	improve scopolamine-induced memory impairment	ACh	↑	[110]
Rats treated with OKA	antioxidative anti-inflammatory	Nrf2	↑	[112]

**Table 6:** Preclinical studies of moringin-mediated neuroprotective effects.

Model	MG-Mediated Protective Effects	Proposed Mechanisms Involved	Up/Down	References
	***In vivo***			
AF64A rats	antioxidative	SOD, CAT MDA, AChE	↑ ↓	[119]
Rats infused with colchicine	ameliorating cognitive functions	SOD, CAT	↑	[120]

**Table 7:** Preclinical studies of morphine-mediated neuroprotective effects.

Model	MOR-Mediated Protective Effects	Proposed Mechanisms Involved	Up/Down	References
	***In vitro***			
Aβ-exposed rat primary neurons	anti-amyloidogenic	Hsp70	↑	[157]
Aβ-primary cortical neurons	anti-amyloidogenic	mTOR	↓	[158]
	***In vivo***			
APP/PS1 double transgenic AD mice	anti-amyloidogenic	Hsp70	↑	[159]

**Table 8:** Preclinical studies of caffeine-mediated neuroprotective effects.

Model	CAF-Mediated Protective Effects	Proposed Mechanisms Involved	Up/Down	References
	***In vivo***			
THY-Tau22 Transgenic mouse	anti-inflammatory antioxidative	TNF-α, GFAP, MAPK, Nrf2, MnSOD	↓ ↑	[170]
AD transgenic mouse model (Tg APP_swe_)	anti-amyloidogenic	PS1, BACE-1	↓	[171]
APP/PS1 double transgenic AD mice	anti-amyloidogenic	BDNF, TrkB	↑	[173]

**Table 9:** Preclinical studies of nicotine-mediated neuroprotective effects.

Model	NIC-Mediated Protective Effects	Proposed Mechanisms Involved	Up/Down	References
	***In vivo***			
AD rat model	anti-amyloidogenic	BACE-1	↓	[185]
AD transgenic mouse model (Tg APP_swe_)	anti-amyloidogenic	nAchRα7	↑	[186]
Male Wistar rats	improved memory performance	ChAT, VAChT NGF, TrkA	↑	[187]

**Table 10:** Preclinical studies of huperzine A-mediated neuroprotective effects.

Model	HupA-Mediated Protective Effects	Proposed Mechanisms Involved	Up/Down	References
	***In vitro***			
SHSY5Y exposed to H_2_O_2_	antioxidative	NGF, P75^NTR^, MAPK/ERK	↑	[195]
Aβ-exposed cell lines	antioxidative anti-amyloidogenic anti-apoptotic	GPx, CAT, ATP ROS Cleaved-caspase 3	↑ ↓	[196,197] [198]
Mutant APP_swe_ over expression in HEK293 cells	anti-amyloidogenic	PKC	↑	[203]
	***In vivo***			
Aβ-infused rats	anti-amyloidogenic	PKC	↑	[203]
Aβ-infused rats	neurogenesis	MAPK/ERK	↑	[201]
Aβ-infused rats	improved memory performance anti-apoptotic	Bax, p53	↓	[202]

**Table 11:** Preclinical studies of berberine-mediated neuroprotective effects.

Model	BER-Mediated Protective Effects	Proposed Mechanisms Involved	Up/Down	References
	***In vitro***			
Mutant APP_swe_ over expression in H4	anti-amyloidogenic	β-secretase	↓	[218]
Mutant APP_swe_ over expression in HEK293 cells	anti-amyloidogenic	β-secretase ERK1/2	↓ ↑	[219]
Mutant APP_swe_ over expression in Neuro 2A	anti-amyloidogenic	GSK3β	↓	[43]
rat primary astrocytes	antioxidative	PI3-kinase/Akt, HO-1	↑	[220]
Aβ-exposed microglia BV2 cells	anti-inflammatory	MAPK, NF-kB	↓	[220]
	***In vivo***			
AD transgenic mouse model (TgCRND8)	improved learning deficits and long-term spatial memory			[43]

**Table 12:** Preclinical studies of cannabidiol-mediated neuroprotective effects.

Model	CBD-Mediated Protective Effects	Proposed Mechanisms Involved	Up/Down	References
Aβ-stimulated PC12 neuronal cells	anti-amyloidogenic	GSK3β Wnt/β-catenin	↓ ↑	[228]
Aβ-stimulated PC12 neuronal cells	antioxidative anti-apoptotic	ROS, iNOS, NO, Casp3	↓	[230,231]
rat cortical neurons exposed to toxic glutamate	antioxidative anti-apoptotic	NMDA, AMPA and kainate receptor toxicity	↓	[232]
SHSY5Y overexpressing APPswe	anti-amyloidogenic	PPARγ	↑	[233]
	***In vivo***			
Aβ-infused mice	anti-amyloidogenic antioxidative anti-inflammatory	iNOS, NO, MAPK, NFκB, IL-1β	↓	[235]
Aβ-injected rats	anti-inflammatory	PPARγ	↑	[234]

## Abbreviations

The following abbreviations are used in this manuscript:

α-Syn	α-Synuclein
ACh	Acetylcholine
AChE	Acetylcholinesterase
AChEIs	Acetylcholinesterase inhibitors
AD	Alzheimer’s disease
Akt	protein kinase B
AMPA	α-amino-3-hydroxy-5-methyl-4-isoxazolepropionic acid
AMPK	5′ adenosine monophosphate-activated protein kinase activated protein kinase
APP	Amyloid Precursor Protein
APP_swe_	Swedish mutant of the Amyloid Precursor Protein
ARE	Antioxidant Responsive Element
Aβ	Amyloid beta
BACE-1	Beta-site amyloid precursor protein cleaving enzyme 1
BBB	Blood brain barrier
Bax	Bcl-2-associated X protein
BChE	Butyryl-cholinesterase
BER	Berberine
CAF	Caffeine
CBD	Cannabidiol
CCH	Chronic cerebral hypoperfusion
ChAT	Choline acetyltransferase
COX-2	Cyclooxygenase 2
CREB	cAMP-responsive element-binding protein
CUR	Curcumin
DLB	Dementia with Lewy bodies
EGCG	Epigallocatechin 3-Gallate
ERK	Extracellular-signal-regulated kinases
FDA	Food and Drug Administration
FTD	Frontotemporal Dementia
GAL	Galantamine
GCLC	Glutamate cysteine ligase
GFAP	Glial fibrillary acidic protein
GPx	Glutathione peroxidase
GSH	Glutathione
GSK3β	Glycogen synthase kinase 3 β
GST	glutathione-S*-*transferase
HEK293_swe_	HEK293 cells trasnsfected with APP_swe_ variant
5-HT1A	5-hydroxytriptamine1A
HO-1	Heme oxygenase-1
Hsp	Heat shock protein
HupA	Huperzine A
icv	intracerebroventricular
icv-STZ	Intracerebroventricular administration of streptozocin
IL-1β	Interleukin-1beta
IL-6	Interleukin-6
iNOS	Inducible nitric oxide synthase
ITCs	Isothiocyanates
JNK	c-Jun N-terminal kinases
LPS	Lipopolysaccharide
MCP-1	Monocyte Chemoattractant Protein-1
MG	Moringin
MnSOD	Manganese-dependent Superoxide Dismutase
mTOR	Mammalian target of rapamycin
N2a/APP_swe_	Neuro2a cells overexpressing mutant APP_swe_ gene
NFκB	Nuclear Factor Kappa B
NFTs	Neurofibrillary tangles
NIC	Nicotine
NMDA	*N*-methyl-d-aspartate
NO	Nitric oxide
NQO1	NAD(P)H:quinone oxidoreductase 1
Nrf2	Nuclear erythroid-2 related factor
OGD/R	Oxygen-Glucose Deprivation/Reoxygenation
OKA	Okadaic acid
pCBs	Phytocannabinoids
PDD	Parkinson’s disease dementia
PGE2	Prostaglandin E2
PI3K	Phosphatidylinositol 3-Kinase
PKA	Protein kinase A
PKC	Protein kinase C
PPARγ	Proliferator-activated receptor gamma antagonist
PS1	Presenilin-1
PS2	Presenilin 2
RESV	Resveratrol
RIV	Rivastigmine
RNS	Reactive Nitrogen Species
ROS	Reactive Oxygen Species
SIRT-1	Sirtuin-1
SOD	Superoxide dismutase
TNF-α	Tumor Necrosis Factor-alpha
TrkA	Tropomyosin receptor kinase A
TrkB	Tropomyosin receptor kinase B
VAChT	Vesicular ACh transporter
VaD	Vascular Dementia
Δ^9^-THC	Delta-9 tetrahydrocannabinol
